# Impact of Metabolites from Foodborne Pathogens on Cancer

**DOI:** 10.3390/foods13233886

**Published:** 2024-12-01

**Authors:** Alice N. Mafe, Dietrich Büsselberg

**Affiliations:** 1 Department of Biological Sciences, Faculty of Sciences, Taraba State University, Main Campus, Jalingo 660101, Taraba State, Nigeria; mafealice1991@gmail.com; 2 Weill Cornell Medicine-Qatar, Education City, Qatar Foundation, Doha Metropolitan Area P.O. Box 22104, Qatar

**Keywords:** metabolites, foodborne pathogens, cancer, detection methods, public health

## Abstract

Foodborne pathogens are microorganisms that cause illness through contamination, presenting significant risks to public health and food safety. This review explores the metabolites produced by these pathogens, including toxins and secondary metabolites, and their implications for human health, particularly concerning cancer risk. We examine various pathogens such as *Salmonella* sp., *Campylobacter* sp., *Escherichia coli*, and *Listeria monocytogenes*, detailing the specific metabolites of concern and their carcinogenic mechanisms. This study discusses analytical techniques for detecting these metabolites, such as chromatography, spectrometry, and immunoassays, along with the challenges associated with their detection. This study covers effective control strategies, including food processing techniques, sanitation practices, regulatory measures, and emerging technologies in pathogen control. This manuscript considers the broader public health implications of pathogen metabolites, highlighting the importance of robust health policies, public awareness, and education. This review identifies research gaps and innovative approaches, recommending advancements in detection methods, preventive strategies, and policy improvements to better manage the risks associated with foodborne pathogens and their metabolites.

## 1. Introduction

Foodborne pathogens encompass many microorganisms, including bacteria, viruses, fungi, and parasites, capable of contaminating food during production, processing, or storage [1]. When ingested, these microorganisms can cause foodborne diseases, a significant global public health challenge [2]. Pathogens enter food systems through various contamination pathways, such as inadequate hygiene, improper cooking, and cross-contamination [3]. The resulting illnesses range from mild gastrointestinal distress to severe, life-threatening conditions, emphasizing the importance of understanding their biology, transmission, and mitigation [4].

*Salmonella* sp., a genus of Gram-negative, rod-shaped bacteria, is one of the most significant contributors to foodborne illnesses globally. It is implicated in two primary health conditions [5]. The first is salmonellosis, a common gastrointestinal disease marked by symptoms such as diarrhea, fever, and abdominal cramps [6]. This condition often arises from consuming raw or undercooked eggs, poultry, or contaminated water. The second is typhoid fever, caused by *Salmonella typhi*, a more severe and systemic illness characterized by prolonged fever and potential complications, including intestinal perforation [7]. The pathogenicity of *Salmonella* sp. stems from its ability to produce lipopolysaccharides and effector proteins, which facilitate the invasion of host cells and the evasion of immune defenses, thereby posing significant health risks [8].

While most strains of *Escherichia coli* are harmless and play a commensal role in the human gut, certain pathogenic types can cause severe illnesses [9]. One such group is the Shiga toxin-producing *E. coli* (STEC), including the notorious *E. coli* O157 strain. These strains are associated with severe health outcomes such as hemolytic uremic syndrome (HUS), which is characterized by acute kidney failure, hemolytic anemia, and thrombocytopenia [10]. Infections often arise from consuming undercooked beef, raw vegetables, or unpasteurized milk. The Shiga toxin produced by STEC is a potent virulence factor that disrupts protein synthesis in host cells, leading to cell death and systemic complications [11]. This makes these strains particularly dangerous and underscores the need for stringent food safety measures [12].

*Campylobacter* species, particularly *C. jejuni* and *E. coli*, are among the most common bacterial causes of diarrheal diseases worldwide [13]. These spiral-shaped, Gram-negative bacteria are typically transmitted through the consumption of undercooked poultry, contaminated water, or raw milk [14]. Infections with *Campylobacter* sp. lead to gastrointestinal symptoms such as diarrhea, abdominal cramps, and fever [15]. Additionally, they can result in autoimmune complications like Guillain–Barré syndrome, a rare but serious condition which affects the nervous system. The bacteria’s ability to colonize the intestines and evade the immune response is facilitated by their flagella and adherence factors, highlighting the importance of prevention strategies in food handling and preparation [16].

*Listeria monocytogenes*, a Gram-positive bacterium, is the causative agent of listeriosis, a severe foodborne illness which poses significant risks to vulnerable populations, including pregnant women, newborns, and immunocompromised individuals [17]. This pathogen is frequently associated with ready-to-eat foods like deli meats, soft cheeses, and raw produce. Listeriosis can manifest in severe forms, such as meningitis and septicemia, and may lead to pregnancy complications, including fetal loss [18]. Notably, *Listeria* sp. can survive and even grow at refrigeration temperatures, making it a persistent challenge in food safety management [19]. This unique trait underscores the critical need for stringent monitoring and control measures in the food supply chain [20].

Mycotoxins are toxic secondary metabolites produced by fungi, including *Aspergillus* sp., *Penicillium* sp., and *Fusarium* sp., which contaminate a wide range of food crops [21]. These compounds pose significant health risks to humans and animals. Among the most concerning mycotoxins are aflatoxins, including *Aspergillus flavus* and *A. parasiticus* [22]. These compounds are highly carcinogenic and are strongly associated with liver cancer, particularly in populations consuming contaminated grains and nuts. Fumonisins, produced by *Fusarium* sp., are linked to esophageal cancer and neural tube defects [23]. Another critical group, ochratoxins, produced by *Aspergillus* sp. and *Penicillium* sp., are nephrotoxic and suspected carcinogens. These mycotoxins often contaminate crops such as grains, nuts, and spices, underscoring the need for stringent food safety measures to mitigate their impact [24].

Mycotoxins, produced as secondary metabolites by certain fungi, have been well-documented for their pathogenic effects on humans and animals, primarily through chronic exposure and their potential carcinogenic, immunosuppressive, and hepatotoxic properties [25]. In contrast, foodborne pathogens secrete toxins that are typically proteinaceous in nature, such as enterotoxins and neurotoxins, [26]. These protein-based toxins operate via distinct pathogenic mechanisms, often causing acute illnesses by directly interacting with host cellular pathways, highlighting a fundamental difference in their modes of action [26]. Mycotoxins are secondary metabolites produced by specific fungi, such as *Aspergillus* sp., *Fusarium* sp., and *Penicillium* sp., often contaminating crops and stored foods under certain environmental conditions [27]. These compounds are chemically diverse, non-proteinaceous, and exhibit chronic toxic effects, including carcinogenicity, hepatotoxicity, immunosuppression, and neurotoxicity [28]. In contrast, toxins produced by foodborne pathogens, such as *Clostridium botulinum*, *Escherichia coli*, and *Staphylococcus aureus*, are primarily protein-based exotoxins [29]. These toxins are typically synthesized during pathogen growth in food or the host, leading to acute diseases through specific interactions with host cellular targets, such as disrupting membranes, inhibiting protein synthesis, or overstimulating immune responses [30]. Thus, mycotoxins and pathogen-derived toxins differ fundamentally in their origins, chemical nature, and mechanisms of pathogenicity [31].

Bacterial toxins are potent substances produced by certain pathogenic bacteria that exacerbate illness and contribute to systemic complications [32]. Shiga toxins, produced by Shiga toxin-producing *Escherichia coli* (STEC), inhibit protein synthesis in host cells, resulting in cell death and conditions such as hemolytic uremic syndrome (HUS), which can cause acute kidney failure [33]. Enterotoxins, secreted by bacteria like *Staphylococcus aureus* and *Bacillus cereus*, are common culprits in acute food poisoning [34]. These toxins lead to symptoms such as vomiting and diarrhea, typically caused by ingesting contaminated foods. Additionally, while not a traditional foodborne pathogen, *Helicobacter pylori* produces toxins that play a role in chronic gastritis and are implicated in gastric cancer, emphasizing the diverse impacts of bacterial toxins on human health [35].

Foodborne pathogens and spoilage organisms also produce other harmful metabolites that impact health. Biogenic amines, such as histamine, can accumulate in spoiled fish, cheese, and fermented foods, triggering toxic reactions in sensitive individuals [36]. The decarboxylation of amino acids forms these compounds during microbial activity. Another critical group of metabolites is lipopolysaccharides (LPSs), components of the outer membrane of Gram-negative bacteria [37]. LPSs can trigger systemic inflammation when they enter the bloodstream and are associated with chronic conditions, including cancer [38]. These metabolites highlight the complexity of food safety issues and the need for comprehensive monitoring and mitigation strategies [39].

Studying foodborne pathogens and their metabolites is critical for identifying health risks and developing mitigation strategies. Many metabolites, especially those with carcinogenic potential, persist in food products even after pathogens are eliminated [40]. This underscores the necessity for advanced detection technologies, such as mass spectrometry and molecular assays, and robust food safety practices [41]. Effective management strategies, including improved hygiene, thorough cooking, and regular food safety monitoring, are essential to reducing exposure and safeguarding public health [42].

The purpose and significance of this article lie in exploring the intricate relationship between these metabolites and cancer, particularly their role in inducing or exacerbating cancer in patients or causing severe complications. Understanding how metabolites like aflatoxins, fumonisins, and bacterial toxins interact with cellular mechanisms to promote carcinogenesis or hinder cancer treatment is vital for developing targeted interventions. Moreover, these metabolites not only pose direct threats to cancer patients but also represent a broader public health challenge by increasing the burden of cancer and related diseases globally.

This overview highlights the multifaceted risks posed by foodborne pathogens and their metabolites, emphasizing their potential to deteriorate the health of cancer patients and the far-reaching implications for public health. It underscores the importance of continued research and preventive measures to mitigate their global impact and improve food safety.

## 2. Metabolites from Foodborne Pathogens

### 2.1. Types of Pathogens and Their Metabolites

Foodborne pathogens such as *Salmonella*, *Campylobacter*, *Escherichia coli*, and *Listeria monocytogenes* are notorious for their ability to cause widespread foodborne illnesses [43]. These microorganisms produce various metabolites during their growth and metabolic processes that can affect human health [44]. For example, *Escherichia coli* (mainly *E. coli* O157) produce Shiga toxins, which cause severe gastrointestinal distress and can lead to hemolytic uremic syndrome (HUS) [45]. *Salmonella* sp. releases endotoxins, contributing to inflammation, fever, and diarrhea. *Listeria monocytogenes*, while primarily known for causing listeriosis, can also produce toxins that lead to significant immune system challenges [5]. *Campylobacter*, a leading cause of bacterial food poisoning, produces metabolites that can trigger gastrointestinal disorders and, in severe cases, neurological conditions like Guillain–Barré syndrome [46].

### 2.2. Metabolites of Concern

Several metabolites produced by these pathogens are associated with severe health conditions, including cancer [47]. For instance, mycotoxins produced by fungi, such as aflatoxins, are potent carcinogens linked to liver cancer [48]. Additionally, bacterial pathogens can generate harmful metabolites, such as the cytolethal distending toxin (CDT) produced by *Campylobacter*, which has been shown to damage DNA and promote tumor development [49]. *Helicobacter pylori*, a bacterium often associated with contaminated food and water, produces urease, which facilitates chronic inflammation in the stomach lining, eventually leading to gastric cancer [50]. Understanding these metabolites is crucial because their impact on health can be significant, contributing not only to acute illnesses but also to long-term, life-threatening diseases like cancer [51].

## 3. Emerging Metabolites and Their Carcinogenic Mechanisms

The field of foodborne pathogens is continuously evolving, and new evidence is surfacing on the roles metabolites from these microorganisms may play in human health, particularly in cancer development. Most research on foodborne pathogens has traditionally focused on gastrointestinal illnesses. Still, a growing body of work is uncovering how specific metabolites might act as carcinogens or co-factors in cancer progression. This is an exploration of metabolites from under-researched foodborne pathogens like *Cronobacter sakazakii* and *Aeromonas hydrophila* and their potential role in carcinogenesis [1]. This study also delves into how the combined effects of multiple pathogen metabolites can exacerbate cancer progression.

### 3.1. Metabolites from Emerging or Under-Researched Foodborne Pathogens

#### 3.1.1. *Cronobacter sakazakii* Metabolites and Their Carcinogenic Mechanisms

*Cronobacter sakazakii* is a Gram-negative pathogen known for causing severe infections, particularly in infants. Traditionally linked to necrotizing enterocolitis, bacteremia, and meningitis in newborns, emerging research points to the ability of *C. sakazakii* to produce specific metabolites that may have carcinogenic effects, especially in gastrointestinal tissues [52].

Lipopolysaccharides (LPSs): LPS, an endotoxin found in the outer membrane of Gram-negative bacteria like *C. sakazakii*, can trigger chronic inflammation in the gastrointestinal tract. Chronic inflammation is a well-documented risk factor for colorectal cancer, as it promotes DNA mutations causing cellular damage and creates an environment conducive to tumorigenesis [53].

Toxins and Secondary Metabolites: Studies have suggested that certain toxins and secondary metabolites produced by *C. sakazakii* could have genotoxic effects, although the precise mechanisms remain largely unstudied. These metabolites may impair DNA repair mechanisms, allowing for the accumulation of mutations which can initiate cancerous growths [54].

Given the rising incidence of *C. sakazakii* infections in adults and its potential ability to colonize the intestines, exploring its metabolites’ carcinogenic effects in adults, especially those with pre-existing inflammatory conditions, represents a novel area of research [55].

#### 3.1.2. *Aeromonas hydrophila* Metabolites and Their Carcinogenic Mechanisms

*Aeromonas hydrophila* is another Gram-negative pathogen commonly found in aquatic environments and associated with foodborne infections from contaminated water, seafood, or produce. While primarily linked to gastrointestinal diseases, its metabolites are increasingly being studied for their role in cancer progression [56].

Aerolysin: *A. hydrophila* produces a pore-forming toxin called aerolysin, which disrupts the intestinal epithelium’s integrity, causing damage and inflammation. Chronic damage to epithelial tissues is a recognized precursor to cancer, as it can lead to dysplasia and the eventual transformation of cells into a neoplastic state.

Siderophores and Reactive Oxygen Species (ROS): Another mechanism by which *A. hydrophila* metabolites may contribute to carcinogenesis is by producing siderophores and iron-chelating compounds. Siderophore activity can promote the generation of ROS, which leads to oxidative stress and DNA damage. Persistent DNA damage without efficient repair can result in mutations and cancer [57].

Endotoxins: Like *C. sakazakii*, *A. hydrophila* also produces endotoxins that can induce a pro-inflammatory response, particularly in the gastrointestinal tract. The relationship between chronic inflammation and cancer development, especially in the context of gastrointestinal cancers, underscores the importance of studying these endotoxins further [58].

Although the direct involvement of these metabolites in cancer remains underexplored, *A. hydrophila* is a promising subject for future investigations due to its capacity to disrupt gut homeostasis and promote a pro-inflammatory environment that could potentially contribute to cancer progression [59].

#### 3.1.3. Synergistic Effects of Multiple Pathogen Metabolites on Cancer Progression

The human body is frequently exposed to multiple pathogens simultaneously, particularly in foodborne illnesses where various microorganisms may contaminate the same food source. Combined exposure to metabolites from different pathogens may exacerbate the carcinogenic potential of individual metabolites through synergistic mechanisms, including increased inflammation, oxidative stress, and DNA damage [60].

- Enhanced Inflammatory Responses

Metabolites from different pathogens, such as LPSs from *C. sakazakii* and aerolysin from *A. hydrophila*, could act in concert to amplify inflammatory responses in the host. Chronic inflammation is one of the critical drivers of cancer, especially in tissues like the gastrointestinal tract, where many foodborne pathogens exert their effects [61].

Cytokine Storms: The interaction between LPSs and other bacterial toxins can produce excessive pro-inflammatory cytokines, such as TNF-α, IL-6, and IL-1β. Sustained high levels of these cytokines promote a cycle of cellular injury, proliferation, and repair that can lead to mutations and malignant transformation [62].

Macrophage Polarization: Some pathogen metabolites influence the polarization of macrophages towards a pro-tumorigenic M2 phenotype. When exposed to multiple metabolites, macrophages may be more likely to adopt this tumor-supporting role, enhancing the growth and survival of cancer cells [63].

- Increased Oxidative Stress and DNA Damage

Oxidative stress is a critical factor in cancer development. Metabolites from foodborne pathogens can contribute to the accumulation of ROS, which causes oxidative damage to DNA, proteins, and lipids. When multiple pathogens are present, the total ROS burden can be significantly higher [64].

Cross-Reaction of Metabolites: For instance, siderophores produced by *A. hydrophila* can facilitate iron uptake in infected tissues, and iron is a well-known catalyst of ROS production. DNA damage can be more severe when combined with LPSs from *C. sakazakii*, inducing an immune response and further oxidative stress. The accumulation of mutations in key oncogenes and tumor suppressor genes (such as p53) can accelerate the initiation of cancer [65].

Metabolic Reprogramming: Certain pathogens may also alter the metabolic landscape of infected cells, making them more susceptible to oxidative stress. For instance, metabolite-induced metabolic shifts toward glycolysis (the Warburg effect) can increase the vulnerability of cells to ROS, compounding the risk of malignant transformation [66].

- Disruption of Gut Microbiota and Epithelial Barrier Integrity

Another synergistic effect arises from the combined impact of pathogen metabolites on the gut microbiota and the intestinal epithelial barrier. Many foodborne pathogens, including *C. sakazakii* and *A. hydrophila*, can alter the gut microbiome’s composition, leading to dysbiosis, which is closely linked to cancer [67].

Microbiome Dysregulation: Pathogen metabolites can shift the balance of the gut microbiota towards pro-inflammatory and carcinogenic bacteria. When multiple pathogens are involved, the disruption is often more severe. This dysbiosis can promote conditions like colorectal cancer by creating a pro-carcinogenic microenvironment that supports tumor growth and suppresses anti-tumor immune responses [68].

Barrier Disruption: Metabolites from multiple pathogens may weaken the gut’s epithelial barrier, increasing permeability (leaky gut). This allows harmful substances, including additional carcinogens from the diet or environment, to enter the bloodstream, where they can reach other tissues and potentially initiate tumorigenesis [69].

#### 3.1.4. Future Directions in Research

Exploring pathogen metabolites and their role in cancer is still in its early stages. Future research should focus on the following:

- Characterizing new metabolites—advances in metabolomics can aid in identifying novel carcinogenic metabolites from under-studied pathogens;
- Longitudinal studies—tracking the long-term effects of chronic exposure to pathogen metabolites and their role in cancer development [70];
- Intervention strategies—investigating probiotic therapies, microbiome modulation, and dietary interventions to mitigate the carcinogenic effects of pathogen metabolites.

Metabolites from emerging or under-researched foodborne pathogens such as *Cronobacter sakazakii* and *Aeromonas hydrophila* hold significant potential to contribute to cancer development through mechanisms such as chronic inflammation, oxidative stress, and microbiome disruption [71]. The synergistic effects of combined pathogen metabolite exposure can further exacerbate these carcinogenic processes. Addressing this emerging risk requires a multifaceted approach involving advanced detection methods, improved food safety measures, and ongoing research into the long-term health impacts of these metabolites [72].

### 3.2. Metabolite Interaction with the Microbiome and Cancer

The gut microbiome, comprising trillions of microorganisms, is crucial in maintaining human health, including regulating immune responses, digestion and even influencing cancer development. When the gut microbiome is disrupted by factors such as metabolites from foodborne pathogens, it can lead to dysbiosis—a state of microbial imbalance, which has been increasingly linked to carcinogenesis. The metabolites produced by these pathogens may alter the composition and function of the gut microbiome, influencing cancer progression through various mechanisms, such as chronic inflammation, disruption of immune homeostasis, and the induction of genotoxicity [51]. This section explores the intricate relationship between foodborne pathogen metabolites and the gut microbiome, emphasizing how these metabolites alter microbial composition, induce dysbiosis, and contribute to cancer development [73].

#### 3.2.1. Microbiome Interaction: How Pathogen Metabolites Disrupt the Gut Microbiota

The gut microbiome is a complex ecosystem; its balance is essential for maintaining health. However, metabolites produced by foodborne pathogens can interfere with the normal microbial flora, promoting an environment conducive to carcinogenesis [74].

- Metabolites and Gut Microbiota Composition

Pathogen metabolites can directly and indirectly affect the microbial community, disrupting the gut’s balance between beneficial and harmful bacteria. Some metabolites produced by foodborne pathogens have the following potential:

They can promote the growth of pathogenic or opportunistic microbes: For instance, metabolites like lipopolysaccharides (LPSs) from Gram-negative bacteria (e.g., *Salmonella*, *Escherichia coli*) can suppress the growth of beneficial bacteria (such as *Lactobacillus* and *Bifidobacterium*) while promoting the proliferation of pathogenic bacteria. This shift in microbial composition can favor pro-inflammatory and tumor-promoting species [75].

They can also have direct antimicrobial activity: Some pathogen-derived metabolites possess antimicrobial properties that may selectively kill beneficial microbes, leading to the loss of microbiota diversity. Reduced diversity in the gut microbiome is a well-established risk factor for diseases such as colorectal cancer [76].

- Influence on Microbial Metabolic Functions

Pathogen metabolites not only alter the composition of the microbiome but also affect the metabolic functions of resident microbes.

Short-Chain Fatty Acid (SCFA) Production: SCFAs (such as butyrate, acetate, and propionate) are beneficial byproducts of the fermentation of dietary fibers by gut bacteria. SCFAs, particularly butyrate, have protective roles in preventing cancer by maintaining epithelial integrity, suppressing inflammation, and inducing apoptosis in cancer cells. Pathogen metabolites can inhibit SCFA-producing bacteria, resulting in lower SCFA levels, which compromises the protective functions of the gut [77].

Secondary Bile Acid Metabolism: The gut microbiota is critical in converting primary bile acids into secondary bile ones. Some secondary bile ones, such as deoxycholic acid (DCA), are carcinogenic and promote DNA damage in epithelial cells. Pathogen metabolites may induce dysbiosis that enhances the production of these carcinogenic bile acids, contributing to colorectal cancer risk [78].

#### 3.2.2. Metabolite-Induced Dysbiosis: Mechanisms Contributing to Carcinogenesis

Dysbiosis, an imbalance in the microbial ecosystem, is a crucial factor linking pathogen metabolites to cancer. Metabolite-induced dysbiosis disrupts the normal protective functions of the gut microbiome, leading to several pathways of carcinogenesis [79].

- Chronic Inflammation and Immune Dysregulation

One primary mechanism by which metabolite-induced dysbiosis contributes to cancer is chronic inflammation. Inflammation is a double-edged sword: while it is essential for immune defense, chronic uncontrolled inflammation can promote cancer by damaging tissues, causing cellular mutations, and creating a tumor-promoting microenvironment [80].

Lipopolysaccharides (LPSs): LPS, produced by Gram-negative bacteria, is a potent inducer of inflammation by activating toll-like receptor 4 (TLR4) on immune cells. Pathogen-derived LPS induces dysbiosis, leading to an exaggerated immune response, including the release of pro-inflammatory cytokines such as IL-6, TNF-α, and IL-1β. Chronic exposure to these cytokines can promote epithelial damage and create a microenvironment that fosters cancer cell proliferation and survival [81].

Metabolite-induced immune evasion: Dysbiosis can shift immune responses in favor of tumor development by impairing immune surveillance mechanisms. For example, dysregulated gut microbiota resulting from pathogen metabolites can reduce the activity of anti-tumor immune cells, such as cytotoxic T cells, while promoting regulatory T cells (Tregs), which suppress anti-tumor immunity. This creates an immune-tolerant environment that allows cancer cells to evade immune destruction [82].

- Gut Barrier Dysfunction: A Pathway to Carcinogenesis

The gut epithelial barrier is the first defense against pathogenic bacteria and their metabolites. However, when dysbiosis compromises this barrier, pathogen metabolites can penetrate deeper into the tissues, promoting inflammation, immune dysregulation, and DNA damage [83].

Increased Gut Permeability (Leaky Gut): Dysbiosis caused by pathogen metabolites can disrupt tight-junction proteins between epithelial cells, leading to increased intestinal permeability. This condition, often called “leaky gut”, allows toxins, metabolites, and even bacteria to enter the bloodstream. Once in circulation, these substances can affect distant organs, including the liver and colon, leading to systemic inflammation and promoting cancer in these tissues [84].

Genotoxic Effects: Some pathogen metabolites, such as colibactin (produced by certain strains of *Escherichia coli*), are directly genotoxic. Colibactin forms DNA adducts, leading to double-strand breaks in DNA. Dysbiosis that increases the abundance of colibactin-producing bacteria heightens the risk of mutagenesis and cancer development, especially in the colon [85].

- Oxidative Stress and DNA Damage

Pathogen metabolites can exacerbate oxidative stress in the gut, contributing to carcinogenesis through increased DNA damage.

Reactive Oxygen Species (ROS) Production: Certain metabolites from foodborne pathogens stimulate the production of ROS, either by the host immune response or through microbial activity. Chronic exposure to high levels of ROS leads to oxidative damage to DNA, proteins, and lipids, which can initiate carcinogenesis. For example, *Helicobacter pylori*, a well-known carcinogenic pathogen, produces metabolites that increase ROS production, leading to DNA damage and the initiation of gastric cancer [86].

Impaired DNA Repair Mechanisms: Dysbiosis induced by pathogen metabolites can impair the host’s DNA repair mechanisms, making cells more susceptible to accumulating mutations. As a result, mutations in critical oncogenes or tumor suppressor genes (such as p53) can arise, promoting cancer initiation and progression [87].

- Tumor-Promoting Metabolites and Microbial Byproducts

Specific microbial metabolites, particularly those produced by dysbiotic microbiota, have been shown to promote tumor growth directly.

Secondary Bile Acids: Dysbiosis can enhance the production of carcinogenic secondary bile acids, such as deoxycholic acid (DCA), which induce DNA damage, promote chronic inflammation, and stimulate cancerous cell growth in the colon [88].

Polyamines: Pathogen-induced dysbiosis may also increase the production of polyamines, such as putrescine and spermidine, essential for cellular growth. Elevated polyamine levels have been associated with increased cancer cell proliferation, particularly in gastrointestinal cancers [89].

#### 3.2.3. The Crosstalk Between Dysbiosis, Metabolites, and the Tumor Microenvironment

Once dysbiosis is established, pathogen metabolites may continue to interact with the tumor microenvironment, exacerbating cancer progression. These metabolites can modulate the behavior of not only cancer cells but also surrounding stromal cells, immune cells, and blood vessels, influencing tumor growth and metastasis [90].

- Impact on Cancer Cell Metabolism

Dysbiosis-induced pathogen metabolites can alter the metabolic programming of cancer cells, making them more aggressive and invasive.

Warburg Efffect: Dysbiosis can promote metabolic reprogramming in cancer cells, driving them towards glycolysis even in the presence of oxygen (the Warburg effect). This metabolic shift enhances the production of lactate, which acidifies the tumor microenvironment and promotes tumor invasion and immune evasion [91].

- Modulation of Angiogenesis

Specific metabolites from dysbiotic microbiota can also promote angiogenesis (forming new blood vessels), which is critical for tumor growth and metastasis. For instance, dysbiosis can lead to the increased production of pro-angiogenic factors like the vascular endothelial growth factor (VEGF), which supports the formation of blood vessels which supply nutrients to growing tumors [92].

#### 3.2.4. Therapeutic Implications: Targeting Metabolite-Induced Dysbiosis

Given the critical role that metabolite-induced dysbiosis plays in cancer development, several therapeutic strategies are being explored to restore microbial balance and mitigate carcinogenesis [93].

- Probiotics and Prebiotics

Probiotics (live beneficial bacteria) and prebiotics (non-digestible fibers that promote beneficial bacterial growth) can help restore the gut microbiome’s balance [94].

Butyrate-Producing Bacteria: Supplementation with butyrate-producing probiotics can restore SCFA levels, improving epithelial integrity and reducing inflammation. Probiotics such as *Lactobacillus* and *Bifidobacterium* have been shown to reduce tumor-promoting metabolites and restore gut homeostasis [95].

Dietary Interventions: Consuming prebiotics such as inulin and resistant starch can increase SCFA production and improve the gut’s barrier function, counteracting the effects of dysbiosis [96].

- Fecal Microbiota Transplantation (FMT)

FMT involves transferring fecal material from a healthy donor to a patient with dysbiosis. This technique has shown promise in restoring microbial balance and reducing dysbiosis-associated cancer risks. However, the long-term efficacy of FMT in cancer prevention remains under investigation [97].

- Pharmacological Interventions

Emerging pharmacological interventions aim to target specific metabolites or pathways involved in dysbiosis-induced carcinogenesis.

Bile Acid Sequestrants: Drugs that bind and sequester carcinogenic bile acids like DCA may help reduce the risk of colorectal cancer in patients with dysbiosis [98].

Antioxidants: Given the role of ROS in cancer development, antioxidant therapy may help mitigate oxidative stress and prevent DNA damage in individuals with dysbiosis [99]. Table 1 summarizes case studies focusing on emerging metabolites from foodborne pathogens and their carcinogenic mechanisms, highlighting their public health implications.

Metabolite interaction with the microbiome is critical in cancer development, primarily through dysbiosis, chronic inflammation, immune dysregulation, and oxidative stress. Metabolites produced by foodborne pathogens disrupt the delicate balance of the gut microbiota, leading to an environment which promotes carcinogenesis [104]. Therapeutic strategies that restore microbial balance, such as probiotics, prebiotics, and pharmacological interventions, offer promising avenues for mitigating the cancer-promoting effects of dysbiosis. Understanding the intricate relationship between pathogen metabolites and the gut microbiome provides valuable insights into preventing and treating microbiome-associated cancers.

## 4. Cancer Risk Associated with Pathogen Metabolites

### 4.1. Mechanisms of Carcinogenicity

**Figure 1:**
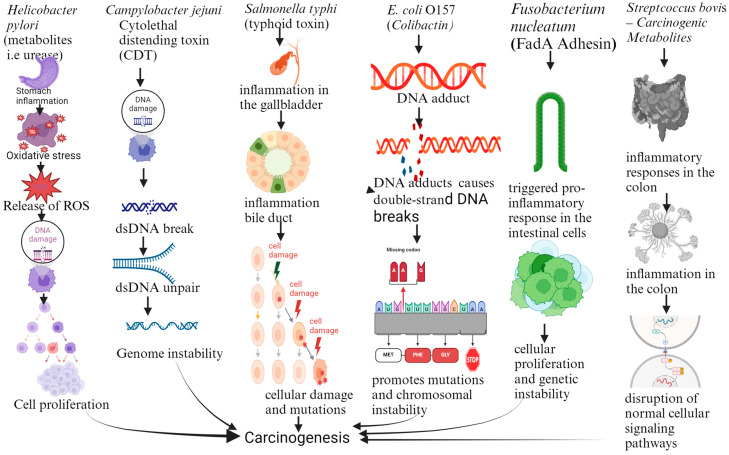
Mechanism of carcinogenicity (created using Bio Render). Legend: ROS: reactive oxygen species; DNA: deoxyribonucleic acid; dsDNA: double-stranded DNA, and CDT: Cytolethal distending toxin.

The metabolites produced by specific foodborne pathogens contribute to cancer development through various biochemical pathways [105]. Pathogens and their metabolites play significant roles in increasing cancer risks through multiple mechanisms. For example, *Aspergillus flavus* produces aflatoxins, mycotoxins which form DNA adducts in liver cells, leading to mutations which disrupt normal cellular processes [73]. These mutations accumulate over time, initiating carcinogenesis and significantly elevating the risk of liver cancer [106]. Similarly, *Helicobacter pylori* produces urease, an enzyme which causes chronic inflammation in the stomach lining, resulting in oxidative stress and the release of reactive oxygen species (ROS) [107]. This environment fosters DNA damage and promotes cell proliferation, thereby increasing the risk of gastric cancer. *Campylobacter jejuni* releases cytolethal distending toxin (CDT), which damages the DNA of host cells by causing double-strand breaks; if left unrepaired, these breaks lead to genomic instability, a critical factor in the development of colorectal cancer [108]. Also, *Salmonella typhi* produces typhoid toxin, inducing chronic inflammation in the gallbladder and bile ducts. This inflammation can cause cellular damage and mutations, predisposing cells to become cancerous, thereby heightening the risk of gallbladder cancer. Certain *Escherichia coli* strains (e.g., *E. coli* O157) produce colibactin, which forms DNA adducts and causes double-strand DNA breaks. If unrepaired, this damage promotes mutations and chromosomal instability, contributing to colorectal cancer development. *Fusobacterium nucleatum* employs FadA adhesin to adhere to and invade intestinal cells, triggering a pro-inflammatory response. This persistent inflammation leads to cellular proliferation and genetic instability, which can progress to colorectal cancer. Lastly, *Streptococcus bovis* produces carcinogenic metabolites that stimulate inflammatory responses in the colon and are associated with polyp formation. These metabolites disrupt normal cellular signaling pathways, increasing the likelihood of malignant transformation and colon cancer development (Figure 1). Another significant carcinogenic mechanism is the disruption of normal cell signaling pathways. For instance, some metabolites activate pathways involved in cell survival and proliferation, such as the NF-κB pathway, which is linked to inflammation and cancer progression [109]. Other metabolites interfere with tumor suppressor genes like p53, impairing the body’s natural ability to prevent cancerous growth [110]. These biochemical disruptions are critical to understanding how pathogen-derived metabolites initiate or promote carcinogenesis [111].

### 4.2. Epidemiological Evidence

Numerous studies have linked exposure to pathogen-derived metabolites with an increased cancer risk [112]. One of the most extensively studied examples is the relationship between aflatoxins and liver cancer [113]. Research shows that populations in regions with high levels of aflatoxin contamination, such as parts of sub-Saharan Africa and Southeast Asia, have significantly higher rates of hepatocellular carcinoma [114]. Animal studies have also demonstrated the carcinogenic effects of aflatoxins, providing further evidence of their cancer-causing potential [115].

*Helicobacter pylori* is a significant focus of epidemiological research for bacterial metabolites. Chronic infection with this pathogen has been strongly associated with gastric cancer, and it has been classified as a Group 1 carcinogen by the International Agency for Research on Cancer (IARC) [116]. Studies in both human and animal models have confirmed the link between *H. pylori* infection, inflammation, and the development of gastric cancer [117]. In terms of *Campylobacter* sp. and its metabolites, although more research is needed, preliminary studies suggest that its CDT may play a role in colorectal cancer development, particularly in regions with high rates of foodborne infections [118].

Together, these studies highlight the significant cancer risk posed by pathogen-derived metabolites, underscoring the need for better detection and control measures to reduce exposure and protect public health [119].

To better analyze and demonstrate the direct impact of foodborne pathogen metabolites on public health, several case studies can be further explored in terms of their emerging metabolites, carcinogenic mechanisms, and public health concerns:

*Cronobacter sakazakii* in Infant Formula: *Cronobacter sakazakii*, a pathogen commonly associated with powdered infant formula, produces metabolites that alter gut permeability and trigger inflammatory responses [120]. These changes can disrupt the gut’s protective barrier, allowing harmful substances to enter the bloodstream and potentially lead to carcinogenesis [121]. In infants, whose immune systems and gut microbiomes are still developing, this disruption can have long-term health implications, increasing the risk of developing cancers later in life due to chronic inflammation and altered immune responses [122]. This case study highlights the vulnerability of infants to pathogen-induced gut dysfunction and the potential for increased cancer risk, especially in populations with compromised gut health.

*Aeromonas hydrophila* in Aquatic Foods: *Aeromonas hydrophila* is a pathogen found in aquatic foods, producing aerolysin and other toxins capable of inducing apoptosis (programmed cell death) and disrupting cellular signaling pathways [123]. These disruptions can promote tumorigenesis, particularly in tissues exposed to these toxins, such as those in the gastrointestinal system [124]. The ingestion of contaminated aquatic products can lead to gastrointestinal cancers, as the toxins alter normal cellular processes and trigger cancer-promoting pathways [125]. The public health concern here is the potential for widespread exposure to this pathogen through contaminated seafood, leading to a significant burden of gastrointestinal cancers [126].

*Fusarium* sp. Mycotoxins in Cereals: Fusarium species, such as *Fusarium graminearum*, produce mycotoxins like zearalenone and deoxynivalenol (DON) in contaminated cereals [127]. These mycotoxins have estrogenic properties, mimicking the effects of the hormone estrogen, and they can cause DNA damage in human cells [128]. The estrogenic activity of these metabolites has been linked to hormonal cancers, such as breast and ovarian cancer, by altering hormonal balances and inducing mutations in cells [129]. Long-term consumption of contaminated grains, especially in regions where food safety regulations may be less stringent, increases the risk of developing these cancers [130]. This case study emphasizes the need for the robust monitoring of food products to mitigate exposure to these harmful metabolites.

*Bacillus cereus* in Rice and Grains: *Bacillus cereus*, a pathogen commonly associated with foodborne illness from rice and grains, produces toxins like cereulide and enterotoxins which induce oxidative stress and DNA damage in human cells [131]. Oxidative stress is a known factor in the initiation of carcinogenic pathways, and the DNA damage caused by these toxins can lead to mutations that promote cancer development [125]. This pathogen poses a particular public health concern in areas where rice and grains are staple foods, and frequent food poisoning incidents increase the risk of gastrointestinal cancers over time [132]. Public health measures aimed at reducing contamination levels in these foods are essential to mitigate these risks [133].

*Clostridium botulinum* in Canned Foods: *Clostridium botulinum*, known for causing botulism through its production of neurotoxins in improperly canned foods, presents an unusual but significant carcinogenic mechanism [134]. The neurotoxins cause severe cell damage and inflammation in affected tissues, which, over time, can contribute to the development of cancer [135]. Although botulism itself is an acute condition, the prolonged effects of neurotoxin-induced inflammation and cellular damage can increase the risk of cancer in affected individuals [136]. This case study underscores the severe public health risks posed by foodborne botulism, highlighting the long-term effects of botulinum toxin exposure that go beyond immediate neurological symptoms and could lead to cancer development over time.

These case studies demonstrate how foodborne pathogen metabolites can contribute to cancer development, from inducing cellular damage and inflammation to disrupting hormonal and immune systems. Each case highlights the need for improved food safety measures, better detection of pathogen contamination, and increased public health awareness, especially in vulnerable populations exposed to these pathogens.

## 5. Detection Methods and Analytical Techniques

### 5.1. Detection Methods

Detection and control strategies for pathogen metabolites focus on identifying harmful metabolites produced by foodborne pathogens and implementing measures to mitigate their impact on human health. These metabolites, often linked to various health risks, including cancer, need to be carefully monitored and controlled to ensure food safety [1].

The detection of pathogen metabolites involves several advanced techniques. Analytical methods such as liquid chromatography–mass spectrometry (LC-MS), gas chromatography (GC), and enzyme-linked immunosorbent assay (ELISA) are widely used to detect and quantify these harmful substances in food samples. In addition, electrochemical and optical biosensors provide rapid and sensitive detection, allowing for the timely identification of specific metabolites. Spectroscopy techniques, such as nuclear magnetic resonance (NMR) and UV–visible (UV-Vis) spectroscopy, help analyze the chemical structures of these metabolites, further enhancing the detection process [137].

Control strategies aim to prevent or minimize the presence of pathogen metabolites in food. Traditional food preservation techniques, such as refrigeration, pasteurization, and drying, inhibit microbial growth and reduce the production of harmful metabolites. Using natural and synthetic antimicrobial agents and bacteriophages can further prevent or decrease the activity of pathogens responsible for producing these metabolites. Probiotics, beneficial microorganisms introduced into food systems, can also out-compete harmful pathogens, reducing their ability to produce toxic compounds [138].

**Figure 2:**
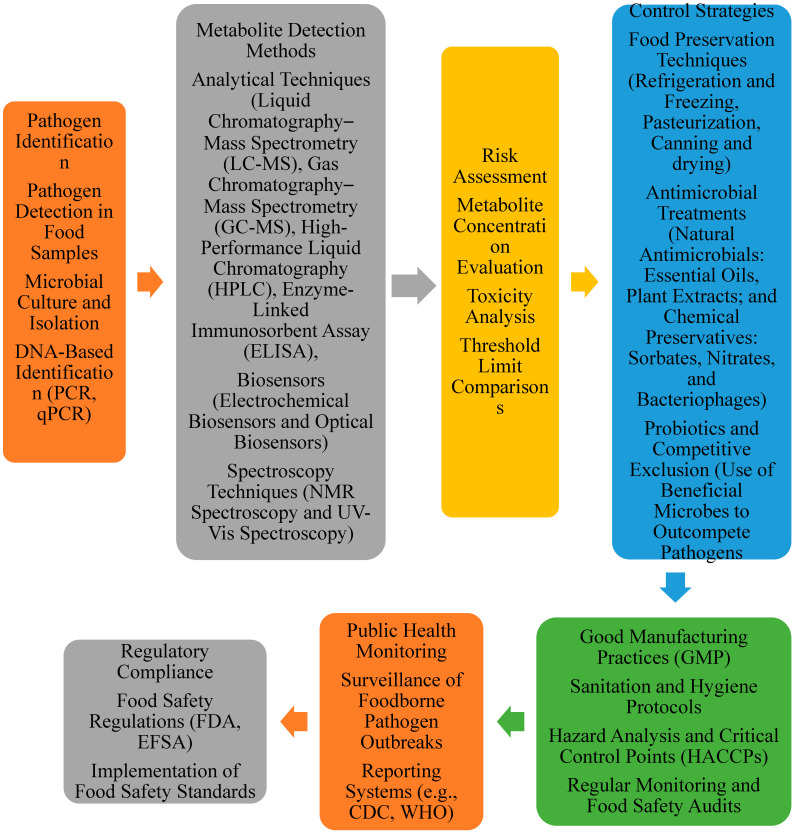
Comprehensive methods for pathogen detection and metabolite identification in food samples: from initial detection to regulatory compliance (created using Microsoft Word version 10.0).

In addition to these methods, maintaining good manufacturing practices (GMPs) is crucial. Ensuring the proper hygiene, handling, and storage of food products reduces contamination risks. In contrast, regulatory compliance with food safety standards, such as hazard analysis and critical control points (HACCPs), ensures consistent monitoring and the prevention of metabolite contamination. These comprehensive strategies are essential in safeguarding public health by reducing exposure to carcinogenic or harmful metabolites from foodborne pathogens (Figure 2) [139].

### 5.2. Analytical Techniques

The detection and quantification of pathogen metabolites in food and biological samples rely on a variety of sophisticated analytical techniques [140] as presented in Table 2.

#### Advantages and Disadvantages of Analytical Techniques

Chromatography offers several advantages for analyzing pathogen metabolites in food and biological samples, particularly techniques like high-performance liquid chromatography (HPLC) and gas chromatography–mass spectrometry (GC-MS). These methods provide high sensitivity, specificity, and the ability to separate complex mixtures, making them ideal for detecting trace levels of carcinogenic metabolites produced by foodborne pathogens [143]. Chromatography also allows for the accurate quantification and identification of multiple metabolites simultaneously, providing valuable insights into their potential health impacts, including cancer risk [144]. However, the disadvantages include needing specialized equipment and trained personnel, which can be costly and time-consuming. Additionally, sample preparation is often labor-intensive, and the techniques may struggle with detecting volatile or low-abundance metabolites [145]. Despite these challenges, chromatography remains a powerful tool in assessing food safety and understanding the carcinogenic potential of pathogen metabolites, ultimately contributing to better public health protection by identifying and mitigating cancer risks [146].

Mass spectrometry (MS) offers significant advantages as an analytical technique for detecting pathogen metabolites in food and biological samples, particularly in assessing cancer risk [147]. It provides high sensitivity, precision, and the ability to identify metabolites at very low concentrations, making it suitable for detecting carcinogenic compounds produced by foodborne pathogens [148]. MS also allows for identifying complex metabolites, providing valuable insights into their biochemical pathways and potential health impacts [149]. However, the disadvantages include the high cost of equipment and maintenance, as well as the need for skilled operators and extensive sample preparation [150]. Mass spectrometry can be challenging in interpreting complex data, mainly when metabolites are present in trace amounts or have similar molecular weights [151]. Despite these challenges, MS remains an essential tool for assessing food safety and understanding the carcinogenic potential of pathogen metabolites, thereby aiding in cancer risk evaluation and public health protection [152].

Immunological assays, such as enzyme-linked immunosorbent assays (ELISA), are valuable for detecting pathogen metabolites in food and biological samples, particularly for assessing cancer risk [153]. One of the key advantages is their high specificity, as they can be designed to target specific metabolites or toxins produced by foodborne pathogens, enabling precise detection [154]. These assays are relatively cost-effective, easy to perform, and can be adapted for high-throughput screening, making them suitable for large-scale food safety testing [155]. However, the main disadvantages include their lower sensitivity compared to other techniques like mass spectrometry, which may limit their ability to detect metabolites at trace levels [156]. Additionally, immunological assays require the availability of high-quality antibodies, which can be challenging to produce for less-studied metabolites [157]. Furthermore, cross-reactivity with other substances in complex food matrices may lead to false positives or inaccurate results, impacting their reliability in assessing cancer-related metabolites [158]. Despite these limitations, immunological assays remain an essential tool for food safety monitoring and the evaluation of potential cancer risks posed by pathogen metabolites [159].

### 5.3. Novel Detection and Analytical Techniques

Detecting and analyzing carcinogenic metabolites from foodborne pathogens, particularly at trace levels, is crucial for the early diagnosis, prevention, and control of cancer development. Recent advances in technology have enabled researchers and clinicians to identify and quantify these harmful metabolites with higher sensitivity, accuracy, and efficiency [160]. Two significant areas of development in this field are advanced detection technologies and metabolomics. These approaches allow for the better detection of carcinogenic metabolites and facilitate biomarker discovery for early cancer diagnosis. This section provides an exhaustive overview of these emerging detection and analytical techniques, with a focus on biosensors, microfluidics, AI-assisted technologies, and the application of metabolomics for cancer biomarker discovery [161].

#### 5.3.1. Advanced Detection Technologies: Emerging Tools for Trace-Level Metabolite Detection

Detecting carcinogenic metabolites from foodborne pathogens at trace levels is essential for identifying early risks associated with cancer development. Advanced detection technologies, including biosensors, microfluidic systems, and artificial intelligence (AI)-assisted methods, are revolutionizing how researchers detect these metabolites with high specificity and sensitivity. These technologies allow for real-time monitoring, rapid testing, and non-invasive sample analysis [162].

- Biosensors for Carcinogenic Metabolite Detection

Biosensors are analytical devices that convert biological interactions into measurable signals. They have become a powerful tool for detecting carcinogenic metabolites due to their sensitivity, specificity, and ability to detect even trace amounts of target compounds in food and biological samples. Biosensors combine a biological recognition element (such as enzymes, antibodies, or nucleic acids) with a transducer, which converts the biological interaction into an electrical, optical, or thermal signal [163].

- Electrochemical Biosensors

Due to their high sensitivity, electrochemical biosensors are among the most common types for detecting metabolites. They measure changes in electrical currents resulting from the interaction between a target metabolite and the biosensor’s recognition element. These sensors can detect carcinogenic metabolites such as aflatoxins, mycotoxins, and nitrosamines, which are linked to cancer development. For instance, electrochemical biosensors have been developed to detect aflatoxin B1, a carcinogenic metabolite produced by the *Aspergillus* species, at extremely low concentrations in food samples [164].

- Optical Biosensors

These biosensors use light to detect metabolite interactions. Surface plasmon resonance (SPR) and fluorescence-based detection can provide real-time, label-free analysis. Optical biosensors are highly effective in detecting specific pathogen metabolites and can be applied to screen food samples for carcinogenic compounds such as polycyclic aromatic hydrocarbons (PAHs), which are byproducts of cooking processes and associated with an increased risk of cancer [165].

Biosensors have significant potential in food safety and public health surveillance. They are portable, cost-effective, and capable of providing rapid results, making them ideal for detecting carcinogenic metabolites in real-world settings such as food production facilities or healthcare clinics [166].

- Microfluidics for High-Sensitivity Detection

Microfluidic technology involves the manipulation of small volumes of fluids within micro-scale channels. It has emerged as a powerful tool for detecting metabolites due to its ability to integrate multiple analytical processes (e.g., separation, detection, and analysis) in a single platform, often called “lab-on-a-chip” systems. Microfluidic devices can perform complex analyses using minimal sample volumes and reagents, reducing the cost and time required for testing [167].

- Lab-on-a-Chip Systems

These miniaturized systems allow for the high-throughput screening of metabolites from foodborne pathogens. Microfluidic devices can incorporate biosensors, chromatography, or mass spectrometry to detect carcinogenic metabolites precisely. For instance, microfluidic chips have been developed to detect nitrosamines and carcinogenic compounds in preserved meats and processed foods. By using these systems, it is possible to detect multiple metabolites simultaneously, offering a comprehensive analysis of potential cancer risks from foodborne pathogens [168].

- Point-of-Care Diagnostics

Microfluidics also enable the development of point-of-care (POC) diagnostic tools for rapidly detecting carcinogenic metabolites in biological samples (such as blood, urine, or saliva). POC systems can be used in clinical settings to screen patients for early signs of cancer or monitor exposure to harmful metabolites. These devices offer a non-invasive, quick, and cost-effective alternative to traditional laboratory testing, making them highly valuable in both public health and clinical applications [169].

Microfluidics have significant potential for revolutionizing how carcinogenic metabolites are detected, allowing for real-time and on-site testing with high precision and minimal human intervention [170].

- Artificial Intelligence (AI)-Assisted Methods for Detection

Artificial intelligence (AI) and machine learning (ML) algorithms are increasingly integrated into metabolite detection systems to improve accuracy, enhance data analysis, and automate complex workflows. AI-assisted techniques can process large datasets generated from biosensors, microfluidic systems, and metabolomics platforms, identifying patterns and correlations which may not be immediately apparent through traditional analysis [171].

AI-Driven Biosensors: AI can optimize biosensor performance by enhancing signal interpretation and reducing noise. AI algorithms can detect subtle changes in biosensor signals and differentiate between specific metabolites, increasing detection sensitivity. For example, AI-assisted electrochemical biosensors have been used to detect cancer-associated metabolites in biological samples by learning from large datasets and refining detection parameters in real time [172].

Machine Learning in Microfluidics: Machine learning algorithms can process the complex datasets produced by microfluidic systems to improve the detection of carcinogenic metabolites. These algorithms can analyze high-throughput data from microfluidic assays, identifying key cancer-associated biomarkers. Machine learning has also been used to predict metabolite interactions with the gut microbiome, helping researchers understand how metabolite exposure may influence cancer risk [173].

AI-assisted methods allow for the more accurate and faster detection of carcinogenic metabolites, making them invaluable for large-scale public health monitoring and personalized medicine.

#### 5.3.2. Metabolomics and Biomarker Discovery: Identifying Unique Cancer Biomarkers from Pathogen Metabolites

Metabolomics, the comprehensive study of metabolites in biological systems, has become essential for identifying biomarkers linked to diseases, including cancer. By analyzing the full spectrum of metabolites in a sample, metabolomics can provide insights into the metabolic pathways affected by pathogen-derived carcinogens and reveal unique biomarkers for early cancer detection [174].

- Metabolomics for Cancer Detection

Metabolomics involves using advanced analytical techniques, such as mass spectrometry (MS) and nuclear magnetic resonance (NMR) spectroscopy, to profile metabolites in food, biological fluids, or tissues. The application of metabolomics in cancer research has significantly advanced the identification of metabolite biomarkers associated with cancer development and progression [175].

- Mass Spectrometry (MS)-Based Metabolomics

MS is a highly sensitive and accurate method for identifying and quantifying metabolites in complex mixtures. MS-based metabolomics has been used to detect carcinogenic metabolites produced by foodborne pathogens, such as aflatoxins, nitrosamines, and PAHs, which are linked to gastrointestinal cancers. MS can detect these metabolites at trace levels, enabling researchers to track early exposure to these carcinogens and their potential links to cancer development [176].

- Nuclear Magnetic Resonance (NMR) Spectroscopy

NMR provides detailed information about the structure and concentration of metabolites in a sample. NMR-based metabolomics can identify unique metabolic signatures associated with cancer, helping to uncover early disease biomarkers. For example, NMR studies have revealed altered metabolite profiles in the urine of individuals exposed to aflatoxins, which are linked to liver cancer [177].

By leveraging metabolomics, researchers can identify early metabolic changes in individuals exposed to foodborne pathogen metabolites, offering a powerful tool for early cancer detection.

- Biomarker Discovery for Early Cancer Detection

One of the critical applications of metabolomics in cancer research is the discovery of biomarker molecules that indicate the presence of disease. Biomarkers can be used for early diagnosis, disease progression, monitoring, and treatment response prediction. Metabolites from foodborne pathogens can serve as biomarkers if they are uniquely associated with cancer development [178].

- Pathogen-Specific Metabolite Biomarkers

Metabolomics has identified several pathogen-specific metabolites that may serve as biomarkers for cancer. For example, mycotoxins, such as aflatoxin B1, have been found in the blood and urine of individuals with liver cancer. Detecting these metabolites in biological samples could allow for the early screening of populations at risk for cancer due to exposure to contaminated food [179].

- Host Metabolic Response Biomarkers

Besides detecting pathogen-derived metabolites, metabolomics can also reveal host metabolic changes in response to these metabolites. For instance, exposure to carcinogenic metabolites from pathogens may trigger oxidative stress, inflammation, or changes in lipid metabolism. These host responses can produce distinct metabolic signatures that serve as early indicators of cancer [180].

- Integrating Metabolomics with Other “Omics” for Comprehensive Biomarker Discovery

Metabolomics is often integrated with other “omics” approaches to enhance biomarker discovery, such as genomics, proteomics, and transcriptomics. This multi-omics strategy provides a more comprehensive understanding of the molecular mechanisms underlying cancer development and allows for the identification of robust biomarker panels [181].

Multi-Omics Approach: By combining metabolomics with genomics (DNA), transcriptomics (RNA), and proteomics (proteins), researchers can map the complete biological response to carcinogenic metabolites. This holistic approach improves the accuracy and reliability of biomarker discovery, enabling the identification of metabolite biomarkers which are specific to certain types of cancer [182].

Data Integration and AI: AI and machine learning are increasingly used to integrate multi-omics data, identifying complex biomarker signatures which could not be detected through a single approach. These AI-assisted methods allow researchers to discover novel biomarkers more efficiently, improving early cancer detection and personalized treatment strategies [183].

Emerging detection technologies and metabolomics are revolutionizing how carcinogenic metabolites from foodborne pathogens are detected and analyzed. Advanced tools like biosensors, microfluidic systems, and AI-assisted methods allow for the sensitive, rapid, and specific detection of these harmful compounds in food and biological samples [184]. Metabolomics provides a comprehensive platform for identifying unique cancer biomarkers associated with pathogen metabolites, offering critical insights into early disease detection and the molecular mechanisms driving cancer development. These novel analytical techniques have great potential for improving public health, food safety, and cancer prevention.

### 5.4. Challenges in Detection

Despite the effectiveness of these techniques, several challenges remain in detecting pathogen metabolites [185], as shown in Table 3.

These challenges highlight the need for ongoing advancements in detection technologies to improve the accuracy, sensitivity, and cost-effectiveness of pathogen metabolite analysis in food safety and public health [40].

## 6. Control Strategies for Growth and Metabolite Production of Foodborne Pathogens

Foodborne pathogens significantly threaten public health, causing a wide range of illnesses. Understanding the mechanisms of pathogen growth and metabolite production is crucial for developing effective control strategies [190]. This section explores various techniques employed in food processing to minimize the risk of foodborne illness [191].

### 6.1. Prevention and Control Measures

Food processing techniques are vital in controlling foodborne pathogens’ growth and metabolite production. The following table outlines vital techniques and their mechanisms of action [138] (Table 4).

### 6.2. Sanitation Practices

Maintaining a clean and sanitary environment is crucial for preventing foodborne illnesses. Adequate sanitation includes personal hygiene, equipment cleaning, and environmental monitoring. The following table highlights essential sanitation practices [201] (Table 5).

### 6.3. Regulatory Measures

Government regulations and industry standards play a vital role in ensuring food safety. These measures establish food production, handling, and processing guidelines to minimize the risk of foodborne illnesses. The following table outlines vital regulatory measures [205] (Table 6).

### 6.4. Emerging Technologies

Technological advancements offer promising solutions for enhancing food safety and controlling foodborne pathogens. These emerging technologies provide novel approaches to food preservation and pathogen inactivation [206]. The following table explores some of these technologies (Table 7).

#### The Potential and Challenges of Emerging Technologies in the Application of Food Detection and Safety Control

Pulsed Electric Fields (PEF): Pulsed Electric Field (PEF) technology is gaining attention for its ability to inactivate microorganisms and enzymes in food without significantly affecting its sensory and nutritional qualities [221]. The high-voltage electric pulses create pores in microbial cell membranes, effectively killing pathogens and spoilage organisms [222]. PEFs have potential for applications in liquid foods like juices and milk, ensuring microbial safety while retaining freshness [223]. However, challenges include the high initial investment costs and energy requirements and the limited applicability to solid foods [224]. Additionally, optimizing processing parameters for various food matrices and ensuring consistent microbial inactivation remain critical issues [225].

Cold Plasma Technology: Cold plasma technology is an emerging non-thermal method for microbial decontamination, offering a rapid and chemical-free alternative for food safety [226]. The reactive species generated by plasma can destroy bacteria, viruses, and fungi on surfaces and within food. This method holds promise for fresh produce, packaging materials, and ready-to-eat foods [227]. However, challenges include scalability for industrial use, the potential formation of undesirable byproducts, and limited knowledge of its long-term effects on food quality and human health [228]. Further research is needed to optimize plasma exposure conditions and assess regulatory concerns.

Ultraviolet (UV) Light: Ultraviolet (UV) light is widely recognized for its antimicrobial properties, among which is the remarkable UV-C light, which disrupts microbial DNA, rendering pathogens inactive [229]. It is effective for surface decontamination, liquid processing, and air purification in food facilities. The technology is cost-effective and environmentally friendly [230]. However, challenges include its limited penetration depth, which reduces efficacy for turbid liquids or opaque surfaces, and the potential for microbial resistance [231]. UV light can also cause oxidative effects, potentially altering the sensory and nutritional properties of certain foods [232].

Nanotechnology: Nanotechnology offers revolutionary potential in food safety through nanosensors for the real-time detection of pathogens, toxins, and spoilage indicators [233]. Nano-encapsulation enhances the stability and delivery of antimicrobial agents or preservatives [234]. However, challenges stem from the lack of standardized regulatory frameworks and uncertainties about the toxicity of nanomaterials [235]. Ensuring consumer acceptance and addressing public concerns about nanotechnology’s safety and environmental impact on food systems is crucial for widespread adoption.

Phage Therapy: Phage therapy utilizes bacteriophages to target and destroy specific bacterial pathogens in foods. It is highly selective, minimizing disruptions to beneficial microorganisms [236]. This approach is particularly valuable for controlling multidrug-resistant pathogens. Despite its potential, challenges include the need for precise phage–host matching, the risk of bacterial resistance to phages, and the potential regulatory hurdles associated with introducing live viruses into food systems [237]. Ensuring phage stability during storage and distribution also requires further innovation.

Biocontrol Using Probiotics: The use of probiotics as biocontrol agents in food safety focuses on their ability to outcompete harmful microbes through mechanisms like competitive exclusion and the production of antimicrobial compounds [238]. This approach is promising for fermented foods, minimally processed products, and biofilms on food processing equipment. However, challenges include maintaining the viability and activity of probiotics during processing and storage, ensuring strain-specific safety and efficacy, and navigating regulatory approval [239]. Further research is needed to understand the long-term implications of probiotic use in diverse food systems.

These emerging technologies present transformative possibilities for enhancing food safety and quality. However, their practical application requires overcoming significant challenges, including cost, scalability, regulatory approval, and consumer acceptance. A multidisciplinary approach involving research, policy development, and industry collaboration is essential to unlock their full potential.

### 6.5. Control Strategies Using Biotechnology

- Probiotic Intervention

Engineered probiotics or microbiota-based interventions can neutralize the harmful metabolites produced by foodborne pathogens. By restoring gut microbiota balance, these probiotics reduce inflammation, bind to toxins (such as aflatoxins), and prevent the absorption of carcinogenic compounds, thereby mitigating their role in cancer development [240].

- Phage Therapy

Phage therapy uses bacteriophages to target and eliminate foodborne pathogens like *Salmonella* sp. or *Cronobacter sakazakii*. By controlling these pathogens, phage therapy reduces the production of carcinogenic metabolites, lowering the risk of cancer from chronic exposure [241]. Table 8 shows case studies and demonstrates how biotechnology can be applied to mitigate the effects of foodborne pathogen metabolites on cancer development. Probiotic interventions and phage therapy offer innovative, targeted strategies to neutralize or control harmful metabolites, contributing to improved public health outcomes.

A multifaceted approach combining traditional methods (thermal processing, sanitation) with emerging technologies (PEF, cold plasma) and regulatory frameworks (HACCP, FSMA) is essential for controlling foodborne pathogens and their metabolites. These strategies are critical to ensuring food safety and public health [246].

## 7. Public Health Implications of Pathogen Metabolites

Foodborne pathogens not only cause infections but also produce metabolites that can have significant adverse effects on human health [14]. These metabolites, particularly toxins, can exert a range of harmful effects, leading to acute and chronic illnesses [247]. Understanding the impact of these metabolites is crucial for public health interventions and strategies to mitigate the risks associated with foodborne pathogens.

### 7.1. Impact on Public Health

Pathogen metabolites, particularly toxins produced by foodborne pathogens, pose significant risks to human health [248]. These metabolites can be carcinogenic, hepatotoxic, neurotoxic, or immunosuppressive, leading to a range of acute and chronic health conditions [249] (Table 9) while Table 10 presents health policies and education.

The global burden of diseases associated with pathogen metabolites emphasizes the need for improved food safety systems to prevent contamination [257].

**Table 10:** Health policies and education.

Roles	Policies	Description
Role of Health Policies	Health policies play a crucial role in regulating food safety, monitoring contaminant levels, and mitigating the risks of pathogen metabolites.	Regulatory Standards: National and international organizations like the World Health Organization (WHO), the Food and Agriculture Organization (FAO), and the Codex Alimentarius Commission have established maximum permissible levels for contaminants such as aflatoxins, fumonisins, and nitrates in food. These guidelines help ensure that food products meet safety standards before they reach consumers [258]. Surveillance and Monitoring: National agencies, such as the U.S. Food and Drug Administration (FDA) and the European Food Safety Authority (EFSA), monitor the presence of pathogen metabolites in food products and agricultural commodities. Early detection through food surveillance systems allows for a rapid response, including product recalls and public warnings. Food Safety Modernization Act (FSMA): The FSMA in the U.S. emphasizes preventive measures over reactive ones. It mandates hazard analysis, supply chain monitoring, and strict adherence to hygiene protocols to prevent contamination at the source. International Collaborations: Global cooperation through platforms like the Global Foodborne Infections Network (GFN) helps countries share information on foodborne disease outbreaks, improving response times and control strategies [259].
Public Awareness and Education	Education plays a critical role in reducing the risks associated with pathogen metabolites. Increasing public awareness helps prevent and respond early to potential foodborne threats.	Regulatory Standards: National and international organizations like the World Health Organization (WHO), the Food and Agriculture Organization (FAO), and the Codex Alimentarius Commission have established maximum permissible levels for contaminants such as aflatoxins, fumonisins, and nitrates in food. These guidelines help ensure that food products meet safety standards before they reach consumers. Surveillance and Monitoring: National agencies, such as the U.S. Food and Drug Administration (FDA) and the European Food Safety Authority (EFSA), monitor the presence of pathogen metabolites in food products and agricultural commodities. Early detection through food surveillance systems allows for a rapid response, including product recalls and public warnings [258]. Food Safety Modernization Act (FSMA): The FSMA in the U.S. emphasizes preventive measures over reactive ones. It mandates hazard analysis, supply chain monitoring, and strict adherence to hygiene protocols to prevent contamination at the source. International Collaborations: Global cooperation through platforms like the Global Foodborne Infections Network (GFN) helps countries share information on foodborne disease outbreaks, improving response times and control strategies.

#### The Role of Policies and Regulations in Controlling Foodborne Pathogens and Ensuring Food Safety

Effective policies and regulations are critical for managing foodborne pathogens and ensuring food safety. These frameworks establish standards for food production, processing, distribution, and consumption, helping to reduce the incidence of foodborne illnesses [260]. However, the approach to food safety varies significantly across countries and regions due to differences in regulatory priorities, resources, and enforcement mechanisms [261].

- Developed Nations: Comprehensive and Stringent Frameworks

In developed nations, such as the United States, the European Union, and Japan, food safety is governed by comprehensive regulatory systems [262]. For instance, the U.S. Food and Drug Administration (FDA) and the U.S. Department of Agriculture (USDA) enforce stringent standards through programs like the Food Safety Modernization Act (FSMA), which emphasizes preventive controls and risk-based approaches. Similarly, the European Union’s General Food Law mandates traceability across the food chain, ensuring rapid responses to food safety incidents. These policies are supported by advanced technologies, robust laboratory networks, and strict enforcement measures, resulting in better control of foodborne pathogens [260].

- Emerging Economies: Balancing Growth and Safety

In emerging economies, regulatory systems are often less developed, leading to inconsistent enforcement of food safety laws [263]. Countries like India, China, and Brazil are making significant strides by implementing food safety reforms [264]. For instance, China’s Food Safety Law emphasizes improved risk assessment and supervision of high-risk foods [265]. However, challenges such as inadequate infrastructure, limited resources for inspections, and fragmented supply chains hinder effective pathogen control [266].

- Low-Income Nations: Resource Constraints and Informal Markets

Low-income nations face significant challenges in controlling foodborne pathogens due to limited resources, weak enforcement, and a high reliance on informal food markets [267]. Many countries in sub-Saharan Africa and South Asia lack standardized food safety frameworks, increasing vulnerability to foodborne diseases [268]. International organizations like the World Health Organization (WHO) and the Food and Agriculture Organization (FAO) work with these nations to build capacity, promote good agricultural practices, and implement hazard analysis and critical control point (HACCP) systems [269].

- Global Harmonization Efforts

Efforts to harmonize food safety standards globally are essential for managing cross-border food trade and mitigating risks of foodborne illnesses. Organizations such as the Codex Alimentarius Commission provide international guidelines and standards that countries can adopt to improve food safety [270]. Free trade agreements and regional bodies like the European Food Safety Authority (EFSA) and the Association of Southeast Asian Nations (ASEAN) Food Safety Policy also play a role in harmonizing standards and facilitating knowledge exchange.

- Challenges and Future Directions

Despite progress, several challenges remain in aligning food safety policies across regions. Variability in regulatory standards, differing levels of technological adoption, and cultural practices can create gaps in food safety. Furthermore, emerging risks such as climate change, antimicrobial resistance, and new foodborne pathogens require adaptive regulatory frameworks [271].

To address these challenges, governments must invest in infrastructure, enhance international collaboration, and engage stakeholders across the food system. Innovative tools like blockchain for traceability, rapid pathogen detection methods, and public–private partnerships can also strengthen food safety globally [272].

While policies and regulations significantly influence the control of foodborne pathogens, their effectiveness depends on enforcement, resource allocation, and international cooperation. Tailoring these frameworks to the specific needs and challenges of each region is vital for achieving safer food systems worldwide [273].

### 7.2. Public Health Implications in Developing Countries

- Focus on Vulnerable Populations

In developing countries, vulnerable populations face higher risks from carcinogenic metabolites due to poor food safety standards, limited access to clean water, and inadequate healthcare systems. Under-resourced settings often lack effective surveillance for foodborne pathogens and cancer, increasing the burden of disease, particularly among children, the elderly, and low-income communities [274].

- Policy Frameworks

Novel policy frameworks integrating food safety and cancer prevention are critical. These should prioritize strengthening food regulation, improving pathogen monitoring systems, and promoting public awareness. Policies could also support biotech interventions like probiotics and phage therapy, aiming to reduce pathogen exposure and related cancer risks in developing nations [275]. Table 11 shows case studies demonstrating the profound impact of foodborne pathogen metabolites on vulnerable populations in developing countries, where food safety and cancer surveillance are often inadequate. Policy frameworks aimed at improving food safety, integrating cancer prevention, and enhancing public health infrastructure can significantly reduce the risk of carcinogenic exposure and improve health outcomes.

Resolving the public health implications of pathogen metabolites requires coordinated efforts across health policy, education, and food safety regulation [279]. By ensuring that food products are safe for consumption and educating the public on proper handling and prevention practices, the risks posed by these harmful metabolites can be effectively mitigated, leading to improved public health outcomes [280].

### 7.3. Climate Change and Foodborne Pathogen Metabolites

Climate change intensifies the proliferation of foodborne pathogens by creating warmer, more humid conditions that promote microbial growth in food systems. This shift increases the production of carcinogenic metabolites like mycotoxins, nitrosamines, and endotoxins. Rising temperatures and unpredictable weather patterns compromise food storage and safety, leading to more significant contamination risks [281]. As a result, climate change is expected to escalate public health concerns related to foodborne pathogens, particularly in vulnerable regions, contributing to higher cancer risks through prolonged exposure to these harmful metabolites. Table 12 shows the case studies highlighting the link between climate change and the increased proliferation of foodborne pathogens and carcinogenic metabolites. They underscore the need for climate-adaptive food safety measures to prevent future public health crises [282].

### 7.4. Comparison of Metabolite Levels in Different Foods

Different foods harbor varying levels of harmful metabolites produced by pathogens. For instance, aflatoxins in maize and peanuts can reach up to 500 µg/kg and 3000 µg/kg, respectively, posing a significant risk for liver cancer. Wheat often contains deoxynivalenol (DON) from *Fusarium* sp., with levels between 200 and 1200 µg/kg, leading to gastrointestinal issues and potential cancer risk. Though present at lower levels, endotoxins from *Vibrio* sp. and *Salmonella* sp. in shellfish and poultry contribute to gastrointestinal and cancer risks [287]. Effective monitoring and food safety strategies are crucial in managing these risks. Table 13 illustrates varying metabolite levels in different foods and their potential health impacts, highlighting the significance of food safety measures in limiting carcinogenic exposure [12].

## 8. Future Directions in Pathogen Metabolite Control and Public Health

While significant strides have been made in understanding and mitigating the risks associated with foodborne pathogens, the threat posed by their metabolites remains a paramount public health concern [12]. These toxins, produced by bacteria and fungi, can cause a range of acute and chronic illnesses, impacting both individual health and global food security [288]. To effectively protect public health, we must continue to invest in research and innovation to address the challenges posed by pathogen metabolites [289]. This section explores critical research gaps and innovative approaches that hold promise for the future of pathogen metabolite control [290].

### Research Gaps

Despite advances in food safety, there are several areas where further research is needed to enhance the detection and control of pathogen metabolites [291] (Table 14) while the innovative approaches are presented on Table 15.

Resolving the challenges of pathogen metabolites in food safety requires a combination of innovative technologies, better detection methods, and an expanded understanding of how these toxins interact with human health [302]. Ongoing research into probiotics, gene editing, natural antimicrobials, and AI-based systems offers promising solutions to reduce the risks posed by these harmful compounds.

## 9. Conclusions

This review underscores the critical public health challenges posed by pathogen metabolites, which can lead to severe health issues such as cancer, liver damage, neurological disorders, and immunosuppression. The need for advanced detection methods, a better understanding of long-term exposure effects, and effective control strategies is evident. Recommendations include prioritizing research into rapid, field-deployable diagnostic tools and innovative approaches like probiotics and gene editing. Strengthening food safety regulations, enhancing global surveillance, and increasing public education on safe food handling practices are essential to mitigate these risks. We can better protect public health and improve food safety by resolving these gaps and implementing targeted policies.

## Figures and Tables

**Table 1:** Case studies in emerging metabolites and their carcinogenic mechanisms.

Case Study	Emerging Metabolite	Pathogen	Carcinogenic Mechanism	Public Health Concern
*Cronobacter sakazakii* in Infant Formula	Cronobacter Metabolites	*Cronobacter sakazakii*	Alters gut permeability and induces inflammatory responses, potentially leading to carcinogenesis.	Increased cancer risk in infants due to compromised gut health [100].
*Aeromonas hydrophila* in Aquatic Foods	Aerolysin and Other Toxins	*Aeromonas hydrophila*	Induces apoptosis and disrupts cellular signaling pathways, promoting tumorigenesis.	Risk of gastrointestinal cancers from contaminated aquatic products [101].
Fusarium mycotoxins in Cereals	Zearalenone and Deoxynivalenol (DON)	*Fusarium* spp.	Estrogenic activity and DNA damage lead to hormonal cancers.	Long-term consumption of contaminated grains raises cancer risk.
*Bacillus cereus* in Rice and Grains	Cereulide and Other Enterotoxins	*Bacillus cereus*	Induces oxidative stress and DNA damage, potentially triggering carcinogenic pathways.	Associated risk of gastrointestinal cancers due to food poisoning incidents [102].
*Clostridium botulinum* in Canned Foods	Botulinum Neurotoxins	*Clostridium botulinum*	Neurotoxin-induced cell damage and inflammation can facilitate cancer development over time.	Severe public health risks in cases of foodborne botulism, with long-term effects [103].

**Table 2:** Analytical techniques.

1.	Chromatography (HPLC, GC-MS)	High-performance liquid chromatography (HPLC) and gas chromatography–mass spectrometry (GC-MS) are widely used for detecting and separating pathogen metabolites in complex samples. HPLC is particularly effective for analyzing non-volatile metabolites, while GC-MS is valuable for volatile and semi-volatile compounds. Both techniques provide high sensitivity and specificity, allowing for the precise quantification of metabolites such as mycotoxins, bacterial toxins, and secondary metabolites from fungi. These methods are highly regarded for their accuracy in distinguishing different metabolite species.
2.	Spectrometry (MS)	Mass spectrometry (MS), often combined with chromatography (e.g., LC-MS or GC-MS), is a powerful tool for identifying and quantifying metabolites based on their mass-to-charge ratio. MS can analyze even trace levels of pathogen metabolites, making it ideal for detecting low-abundance carcinogenic compounds in food and biological samples. MS techniques can also provide structural information about metabolites, helping to identify specific toxins or degradation products linked to foodborne pathogens [141].
3.	Immunoassays	Immunoassays, such as enzyme-linked immunosorbent assays (ELISA), are widely used to rapidly detect pathogen metabolites, mainly in routine food safety testing. These assays are based on antigen–antibody interactions, where specific antibodies bind to target metabolites, allowing for quick, cost-effective, and large-scale screening. ELISA is commonly used to detect mycotoxins like aflatoxins and bacterial toxins like Shiga toxins in food products [142].

**Table 3:** Challenges in detection.

Challenge	Description
Sensitivity and Specificity	One of the significant challenges in detecting pathogen metabolites is achieving high sensitivity and specificity. Many metabolites occur in extremely low concentrations, especially in complex food matrices or biological samples, making them difficult to detect without advanced methods [186]. False positives or negatives can also arise due to cross-reactivity in immunoassays or inadequate separation in chromatographic methods.
Sample Complexity	Food and biological samples often contain interfering substances, such as fats, proteins, or other organic compounds, which complicate detection. Sample preparation methods, including extraction and purification, must be carefully designed to minimize interference and concentrate the metabolites of interest. However, these processes can be time-consuming and require specialized expertise [187].
Cost and Accessibility	Advanced detection methods like GC-MS or LC-MS are expensive and require skilled technicians and sophisticated laboratory infrastructure. This limits their accessibility, especially in regions where foodborne pathogens are prevalent but resources are scarce [188]. Immunoassays, while more affordable and user-friendly, may lack the same level of precision as chromatographic or mass spectrometric techniques [189].

**Table 4:** Food processing techniques.

Technique	Description
Thermal Processing (Pasteurization and Sterilization)	This involves heating foods to a specific temperature for a set period to kill or inactivate pathogens [192]. Pasteurization is standard in dairy products and juices, reducing pathogens like *Salmonella*, *Escherichia coli*, and *Listeria monocytogenes*. Sterilization, often used for canned goods, involves higher temperatures for complete microbial inactivation [193].
Cold Preservation (Refrigeration and Freezing)	Low temperatures slow or stop microbial growth. Refrigeration below 5 °C prevents pathogen growth, while freezing stops microbial activity. However, freezing does not kill all pathogens, so proper thawing and handling are essential.
Dehydration and Drying	Water activity is a critical factor for microbial growth. Drying methods (e.g., air drying and freeze drying) lower the water content of foods, inhibiting pathogens. They are often used for grains, fruits, and meats [194].
Fermentation	Controlled fermentation using lactic acid bacteria or yeasts creates acidic conditions that inhibit pathogens like *Clostridium botulinum* [195]. Producing organic acids, bacteriocins, and alcohol during fermentation can enhance food safety [196].
Irradiation	Ionizing radiation, such as gamma rays or electron beams, kills pathogens by damaging their DNA. This method is effective for spices, meats, and some produce without raising the temperature.
High-Pressure Processing (HPP)	HPP uses high pressure (up to 600 MPa) to inactivate pathogens without significantly altering the food’s sensory properties [197]. It is effective against bacteria like *Listeria* and *E. coli* in foods like juices and meats [198].
Chemical Preservatives	Organic acids (lactic, acetic) and salts (nitrates, sulfites) are used to control pathogen growth [199]. These agents can directly inhibit microbial enzymes or alter the pH to levels unsuitable for pathogen survival [200].

**Table 5:** Sanitation practices.

Practices	Description
Personal Hygiene	Proper hand washing and the use of protective gear like gloves and masks are essential to prevent food handler contamination. This is especially important in high-risk areas like kitchens and food processing plants and during food preparation [202].
Cleaning and Sanitizing Equipment	Equipment used in food production must be regularly cleaned and sanitized to prevent cross-contamination. Effective sanitization agents include chlorine-based solutions, quaternary ammonium compounds, and peracetic acid [203].
Environmental Monitoring	Monitoring the production environment (e.g., floors, walls, and equipment surfaces) for microbial contamination is crucial. Swabbing and testing for pathogens like *Listeria* sp. can prevent cross-contamination [204].
Segregation of Raw and Cooked Foods	Ensuring that raw and ready-to-eat foods are handled separately can reduce the risk of cross-contamination. This includes separate storage, preparation areas, and utensils for raw and cooked foods.

**Table 6:** Regulatory measures.

Measures	Description
Hazard Analysis and Critical Control Points (HACCPs)	This systematic approach identifies potential hazards in the production process and establishes critical control points (CCPs) to reduce or eliminate risks. HACCPs are mandatory for many food industries worldwide and are widely recognized for their effectiveness in preventing foodborne illnesses.
Good Manufacturing Practices (GMPs)	GMPs provide guidelines for producing, handling, and processing food products. These include proper facility design, sanitation, employee hygiene, and pest control measures to reduce contamination risks.
Food Safety Modernization Act (FSMA)	Enacted in the U.S., FSMA shifts the focus from responding to foodborne illness outbreaks to preventing them. It includes provisions for regular inspections, food safety plans, and increased oversight of imported foods.
Codex Alimentarius Standards	Developed by the FAO and WHO, the Codex provides international food standards, guidelines, and codes of practice to ensure food safety and fair-trade practices. These guidelines help harmonize food safety regulations across countries.

**Table 7:** Emerging technologies.

Technologies	Description
Pulsed Electric Fields (PEFs)	PEF uses short bursts of high voltage to create pores in microbial cell membranes, effectively killing or inactivating pathogens without heating the food. This method is being explored for juices, milk, and liquid eggs [207].
Cold Plasma Technology	This non-thermal technology generates ionized gas (plasma) [208] that contains reactive oxygen and nitrogen species capable of killing bacteria, yeasts, and molds [209]. Cold plasma is studied in fresh produce, meats, and packaging materials [210].
Ultraviolet (UV) Light	UV light at specific wavelengths (particularly UV-C) damages the DNA of pathogens, [211], preventing their growth and reproduction. It is used in surface sanitation, water purification, and air treatment in food processing facilities [212].
Nanotechnology	Nanoparticles, particularly silver and copper [213], are being incorporated into packaging materials and coatings for antimicrobial purposes [214]. These materials can prevent pathogen growth on food surfaces and extend shelf life [215].
Phage Therapy	Bacteriophages (viruses which infect bacteria) are being explored as a targeted method to control particular pathogens like *Listeria* sp. or *Salmonella* sp. in foods [216]. Phages offer a natural and specific approach to pathogen control without affecting beneficial microorganisms [217,218].
Biocontrol Using Probiotics	Using beneficial microbes (probiotics) to outcompete or inhibit pathogens in food is gaining traction [219]. For example, *Lactobacillus* sp. species can inhibit *Listeria* sp. in fermented foods, while certain yeast strains are being investigated for pathogen control in alcoholic beverages [220].

**Table 8:** Case studies of control strategies using biotechnology.

Control Strategy	Case Study	Mechanism of Action	Impact on Carcinogenic Metabolites	Outcome
Probiotic Intervention	Engineered Probiotics for Neutralizing Aflatoxins	Genetically engineered probiotics (*Lactobacillus rhamnosus*) are designed to bind and detoxify aflatoxins in the gastrointestinal tract.	The probiotics bind aflatoxin B1, a carcinogenic metabolite produced by *Aspergillus* sp., neutralizing its effect and preventing absorption into the bloodstream.	Reduction in aflatoxin B1 bioavailability and minimized risk of liver cancer from aflatoxin exposure [242].
Probiotic Intervention	Microbiota-Based Interventions to Prevent Dysbiosis and Carcinogenesis	The introduction of beneficial strains (*Lactobacillus plantarum* and *Bifidobacterium bifidum*) is needed to restore gut microbiome balance and prevent dysbiosis triggered by pathogen metabolites.	These probiotics improve gut integrity, reduce inflammation, and prevent the overgrowth of harmful bacteria that produce carcinogenic metabolites like nitrosamines.	Reduced inflammation and lower risk of gastrointestinal cancer due to balanced microbiota and suppression of harmful metabolite production [243].
Phage Therapy	Phage Treatment to Control *Salmonella* sp. in Food Production	Bacteriophages specific to *Salmonella* sp. are used to target and eliminate the pathogen in food processing environments.	By targeting Salmonella, phage therapy prevents the production of endotoxins and other carcinogenic metabolites produced during infection.	Significant reduction in *Salmonella* sp. contamination, decreasing the risk of cancer from chronic exposure to pathogen-associated toxins [244].
Phage Therapy	Phage-Based Control of *Cronobacter sakazakii* in Infant Formula	Bacteriophages specific to *Cronobacter sakazakii* are used to control contamination in powdered infant formula.	Phage therapy reduces the population of *C. sakazakii*, preventing the production of carcinogenic metabolites which may contribute to long-term health issues such as cancer in infants.	Lower contamination rates in infant formula, leading to reduced cancer risks from early exposure to pathogen metabolites [245].

**Table 9:** Impact on public health.

Impact	Description
Carcinogenic Effects	Aflatoxins (produced by *Aspergillus* sp.) are one of the most potent carcinogens found in food. Chronic exposure, particularly in developing countries where food storage conditions may promote fungal growth, is associated with liver cancer [22]. The burden of aflatoxin-related liver cancer is exceptionally high in sub-Saharan Africa and Southeast Asia, where hepatitis B virus infection is also prevalent, exacerbating cancer risk [250]. N-nitroso compounds (produced during the processing of meats) are linked to colorectal cancer. These metabolites are formed from nitrites and nitrates used in food preservation and are classified as probable human carcinogens by the International Agency for Research on Cancer (IARC) [251].
Hepatotoxicity	Pathogen-derived toxins, like aflatoxins and microcystins (produced by cyanobacteria), can cause severe liver damage. Acute aflatoxicosis can lead to liver failure, while chronic exposure leads to liver cirrhosis and increased susceptibility to liver cancer.
Neurological Disorders	Botulinum toxin (produced by *Clostridium botulinum*) is one of the most potent neurotoxins known. It can cause botulism, a life-threatening illness characterized by muscle paralysis, respiratory failure, and death if untreated [252]. Fumonisins, produced by *Fusarium* sp. in grains, are associated with neural tube defects in populations that consume contaminated maize. Animal studies also suggest a link between fumonisins and esophageal cancer [253].
Immunosuppression	Some pathogen metabolites, such as aflatoxins, have immunosuppressive effects, weakening the body’s ability to fight infections [254]. This makes individuals more susceptible to other diseases, including HIV/AIDS and malaria, particularly in regions where these conditions are prevalent [255].
Burden of Foodborne Illnesses	Foodborne illnesses caused by bacterial pathogens (*Salmonella* sp., *E. coli*, and *Campylobacter* sp.) and their toxins lead to gastrointestinal diseases like diarrhea, which can be fatal in vulnerable populations such as children, the elderly, and immunocompromised individuals [256]. Chronic complications include post-infectious irritable bowel syndrome (IBS) and Guillain–Barré syndrome (a severe neurological disorder).

**Table 11:** Case studies of public health implications in developing countries.

Case Study	Vulnerable Populations Affected	Metabolite Impact in Under-Resourced Settings	Policy Frameworks Proposed	Outcome
Aflatoxin Exposure in Rural Africa	Rural communities are dependent on maize and ground nuts as staple foods.	High levels of aflatoxins in improperly stored crops increase liver cancer risk, with limited cancer screening and food safety regulations.	Development of regional policies for improved crop storage, community education on mycotoxin risks, and integration of cancer surveillance programs.	Reduced aflatoxin-related liver cancer cases and improved early detection rates in rural areas [276].
*Cronobacter sakazakii* in Infant Formula in Southeast Asia	Infants in low-income households are reliant on formula feeding.	Contamination of powdered infant formula with *Cronobacter sakazakii* increases risks of infections and long-term cancer effects, with limited food safety oversight.	Strengthening regulations for infant formula production, implementing routine pathogen testing, and subsidizing safer alternatives for low-income families.	Lower contamination rates and reduced infant mortality and cancer risks from early-life pathogen exposure [100].
Hepatitis B and Mycotoxins in Sub-Saharan Africa	These affect low-income populations with poor access to vaccines and healthcare.	Co-exposure to hepatitis B virus and aflatoxins exacerbates liver cancer risks, with little access to vaccinations or mycotoxin control programs.	Integrating mycotoxin control into national cancer prevention policies, expanding hepatitis B vaccination, and improving food safety through regional cooperation.	Decreased liver cancer incidence due to improved vaccination and food safety measures [277].
Foodborne Bacterial Infections in Latin America	These affect children and the elderly in regions with poor sanitation and food handling practices.	Exposure to bacterial pathogens produces carcinogenic metabolites due to a lack of sanitation and food safety measures, which increase cancer risks.	Implement cross-border food safety policies, including stricter controls on imports and exports, and invest in sanitation infrastructure.	Enhanced food safety and reduced incidence of foodborne diseases leading to cancer [278].

**Table 12:** Case studies of climate change and foodborne pathogen metabolites.

Case Study	Climate Change Impact	Pathogen/Metabolite Affected	Public Health Concern	Outcome
Aflatoxin Contamination in Maize (Sub-Saharan Africa)	Increased droughts and heat stress cause higher fungal contamination in crops, especially maize.	*Aspergillus* sp. produces aflatoxins.	Elevated liver cancer risk due to increased aflatoxin levels in staple crops, particularly affecting rural populations.	This results in an increased incidence of liver cancer and the urgent need for climate-resilient crop storage and fungal monitoring systems [283].
Vibrio Infections in Seafood (Coastal Regions of North America and Europe)	Rising ocean temperatures promote the growth of Vibrio bacteria in shellfish, increasing the risk of bacterial infections.	*Vibrio* sp. produces harmful endotoxins.	Greater risk of gastrointestinal cancers linked to chronic exposure to Vibrio endotoxins through seafood consumption.	This results in a growing number of foodborne illnesses; stricter seafood safety regulations and monitoring are needed [284].
Mycotoxin Production in Wheat (Europe)	Warmer and wetter conditions during the growing season lead to increased fungal contamination in wheat.	*Fusarium* sp. produces deoxynivalenol (DON) and other mycotoxins.	Increased risk of cancers related to chronic mycotoxin exposure through wheat-based products.	Food products have a higher prevalence of mycotoxin contamination; enhanced monitoring and climate-adaptive farming practices are required [285].
Salmonella in Poultry (Global)	Rising temperatures accelerate the proliferation of *Salmonella* sp. in poultry farming and food processing environments.	*Salmonella* sp. produces endotoxins and other carcinogenic compounds.	Increased risk of foodborne illnesses and cancer from chronic *Salmonella* sp. exposure.	This results in higher contamination rates and foodborne illness and the need for enhanced cooling and sanitation measures in poultry farming [286].

**Table 13:** Comparison of metabolite levels in different foods.

Food Item	Pathogen	Metabolite	Average Metabolite Level (µg/kg)	Health Impact
Maize	*Aspergillus* sp.	Aflatoxins	10–500	Liver cancer risk
Wheat	*Fusarium* sp.	Deoxynivalenol (DON)	200–1200	Gastrointestinal issues and cancer
Peanuts	*Aspergillus* sp.	Aflatoxins	20–3000	Hepatocellular carcinoma
Shellfish	*Vibrio* sp.	Endotoxins	0.5–5	Gastrointestinal cancers
Poultry	*Salmonella* sp.	Endotoxins and Nitrosamines	5–50	Increased cancer risk
Dairy Products	*Campylobacter* sp.	Toxins (*Campylobacter jejuni*)	1–10	Gastrointestinal disorders and cancer
Fruits (e.g., Apples)	*Penicillium* sp.	Patulin	10–50	Increased cancer risk
Soy Products	*Streptomyces* sp.	Streptomycin Residues	0.1–10	Disruption of gut microbiota and potential cancer

**Table 14:** Research gaps.

Research Gap	Description
Improved Detection Methods	Rapid, Field-Deployable Detection Tools: Current detection methods for pathogen metabolites, like aflatoxins and fumonisins, often require sophisticated lab equipment (e.g., HPLC, ELISA), making them inaccessible in resource-limited settings. Portable, cost-effective, and easy-to-use diagnostic tools that can be deployed in the field or at small-scale production facilities are needed. This is especially critical in regions with high contamination risks, such as sub-Saharan Africa and Southeast Asia [99]. Biosensors: Developing biosensors that can quickly and accurately detect multiple metabolites (mycotoxins, bacterial toxins, etc.) in food matrices is an important research priority. These devices could provide real-time monitoring of contamination in food processing lines, helping to reduce outbreaks of foodborne illnesses [100]. Early Detection of Contaminants in Supply Chains: Better surveillance systems are needed to detect contaminants at early stages of the food supply chain, such as on farms or during storage. This includes better methods for detecting fungal growth or toxin production in grains before they reach consumers [101].
Mechanisms of Metabolite Toxicity	Understanding Chronic Exposure: While the acute toxicity of some pathogen metabolites is well-documented [102], the long-term effects of chronic low-dose exposure (e.g., through diet) are not fully understood. More research is needed to investigate how prolonged exposure to aflatoxins, fumonisins, or nitrates may contribute to cancers, immune disorders, and developmental issues [103]. Interaction Between Pathogens and Metabolites: The interactions between different pathogens (bacteria, fungi) and the metabolites they produce in complex food matrices need more exploration. Understanding how environmental factors (e.g., humidity, temperature) influence these interactions could help develop better prevention and control measures [104].
Probiotic Interventions	The role of probiotics and the human microbiome in mitigating the effects of pathogen metabolites is an emerging research area [292]. Further investigation is needed into how beneficial microbes [293] (such as *Saccharomyces cerevisiae* var. *boulardii*) can detoxify harmful metabolites or inhibit the growth of toxin-producing pathogens [294].
Food Safety and Climate Change	Climate change alters environmental conditions to promote the growth of toxin-producing pathogens, particularly fungi. Research into how shifting climate patterns affect the production of pathogen metabolites in crops and food products is needed to predict and mitigate future risks [295].

**Table 15:** Innovative approaches.

Approaches	Description
Predictive Analytics and AI in Food Safety	Machine Learning and Artificial Intelligence: AI and machine learning models are being developed to predict foodborne pathogen outbreaks and contamination in supply chains. These models can analyze large datasets (weather patterns, food processing conditions, and shipment routes) to identify trends and risks. Predictive analytics could help food safety regulators and producers proactively prevent contamination before it reaches consumers [109]. Blockchain for Supply Chain Transparency: Blockchain technology enhances traceability and transparency in the food supply chain. It enables the real-time tracking of food products from farm to table, helping detect contamination sources quickly and ensuring accountability in handling practices.
Microbiome-Based Solutions	Recent research into the human microbiome has revealed that certain probiotic strains can help mitigate the effects of pathogen metabolites. For example, some probiotic strains have been shown to bind and neutralize aflatoxins, preventing their absorption in the gastrointestinal tract. This presents a promising avenue for developing functional foods and supplements designed to detoxify harmful metabolites in the body [296].
CRISPR and Gene Editing	Gene editing tools like CRISPR are being explored to target and eliminate toxin-producing genes in pathogens. By removing or disabling specific genes responsible for metabolite production, safer fungi or bacteria may be possible for food production. CRISPR technology could also be used to engineer plants resistant to contamination by pathogens or mycotoxins [297].
Natural Antimicrobials and Toxin Binders	Plant-Based Compounds: Plant-derived compounds such as essential oils (e.g., from oregano and thyme) and bioactive peptides are being researched for their antimicrobial and antifungal properties. These natural antimicrobials could be incorporated into food packaging or coatings to prevent pathogen growth and toxin production during storage [112]. Toxin Binders: Another innovative area of research is the development of bioactive compounds that can bind and neutralize harmful metabolites in foods. Certain clays, for instance, have been found to bind aflatoxins, preventing their absorption when consumed. These binders could be used as food additives or in feed to protect both animals and humans from toxin exposure.
Smart Packaging and Sensors	Smart packaging with embedded sensors that monitor the condition of food products (e.g., temperature, humidity, and microbial activity) is an emerging trend [298]. These sensors can detect changes in the food environment that may promote the growth of pathogens and the production of harmful metabolites. For example, sensors that detect volatile compounds associated with fungal growth could trigger alerts before spoilage occurs [299].
Biopreservation	Biopreservation involves using natural or controlled microbial flora and their metabolites to extend the shelf life of food and inhibit the growth of spoilage and pathogenic microorganisms [300]. Lactic acid bacteria and yeasts can produce organic acids, hydrogen peroxide, and bacteriocins that inhibit harmful microbes. This approach aligns with consumer demand for more “natural” food preservation methods without synthetic additives [301].

## Data Availability

No new data were created or analyzed in this study. Data sharing is not applicable to this article.
